# Identifying Metabolic Syndrome in African American Children Using Fasting HOMA-IR in Place of Glucose

**Published:** 2011-04-15

**Authors:** Sushma Sharma, Robert H. Lustig, Sharon E. Fleming

**Affiliations:** Department of Nutritional Sciences and Toxicology, University of California at Berkeley. Dr Sharma is also affiliated with the Dr Robert C. and Veronica Atkins Center for Weight and Health.; Dr Robert C. and Veronica Atkins Center for Weight and Health, and the Division of Pediatric Endocrinology, University of California at San Francisco, San Francisco, California; Dr Robert C. and Veronica Atkins Center for Weight and Health, and the Department of Nutritional Sciences and Toxicology, University of California, Berkeley, California

## Abstract

**Introduction:**

Metabolic syndrome (MetS) is increasing among young people. We compared the use of homeostasis model assessment of insulin resistance (HOMA-IR) with the use of fasting blood glucose to identify MetS in African American children.

**Methods:**

We performed a cross-sectional analysis of data from a sample of 105 children (45 boys, 60 girls) aged 9 to 13 years with body mass indexes at or above the 85th percentile for age and sex. Waist circumference, blood pressure, and fasting levels of blood glucose, insulin, triglycerides, and high-density lipoprotein cholesterol were measured.

**Results:**

We found that HOMA-IR is a stronger indicator of MetS in children than blood glucose. Using HOMA-IR as 1 of the 5 components, we found a 38% prevalence of MetS in this sample of African American children and the proportion of false negatives decreased from 94% with blood glucose alone to 13% with HOMA-IR. The prevalence of MetS was higher in obese than overweight children and higher among girls than boys.

**Conclusion:**

Using HOMA-IR was preferred to fasting blood glucose because insulin resistance was more significantly interrelated with the other 4 MetS components.

## Introduction

Metabolic syndrome (MetS) is a cluster of the most dangerous risk factors for type 2 diabetes mellitus and cardiovascular disease (CVD). Clinical diagnosis of MetS in adults includes the presence of at least 3 of 5 conditions: elevated triglycerides, low high-density lipoprotein cholesterol (HDL-C), high fasting blood glucose, high blood pressure, and obesity (1). Many professional groups, including the World Health Organization, National Cholesterol Education Program Expert Panel on Detection, Evaluation, and Treatment of High Blood Cholesterol in Adults (Adult Treatment Panel III), International Diabetes Federation (IDF), the American Diabetes Association, and the American Heart Association have offered definitions of MetS for adults, but these definitions cannot be used directly for children. Because MetS incidence is increasing rapidly (2), it is vital to identify MetS during childhood to prevent the progression to CVD and type 2 diabetes in adulthood. Laboratory screening of children for MetS can be an impractical approach, so efforts have been made to develop simple screening criteria to identify children who need further testing. Previous studies have modified the criteria for adults when investigating MetS prevalence in children and adolescents (3-7).

The recent IDF consensus definition for children has been built on these previously published definitions, using sex- and age-specific cut points (8). Even though metabolic diseases may be influenced by race/ethnicity (9), the IDF did not consider racial/ethnic endpoints. Cut points specific to sex, age, and race/ethnicity for body mass index (BMI) and waist circumference (10) have been used to determine the prevalence of MetS in a sample of children aged 13 to 15 years, predominantly African American girls (11). The prevalence of MetS in younger African American girls and in African American boys has not been reported to our knowledge nor has there been a comparison by sex. Being overweight is associated with higher incidence of MetS in adolescents (3,12), but few data are available regarding the prevalence of MetS specifically in overweight and obese African American children.

We aimed to 1) identify the prevalence of MetS in overweight and obese African American boys and girls aged 9 to 13 years living in inner-city Oakland, California, 2) determine whether the prevalence of MetS is higher in obese than in overweight African American children, and 3) compare the discriminating power of fasting blood glucose concentration with that of the homeostasis model assessment of insulin resistance (HOMA-IR) as MetS indicators in African American children.

## Methods

### Study participants

Of the 128 participants enrolled in the summer of 2007, a full set of data was available for 108 African American children who were part of the Taking Action Together Study, a community-based lifestyle modification program to reduce the risk for type 2 diabetes (described more fully elsewhere) (13; http://clinicaltrials.gov/ct2/NCT01039116). Study participants were recruited by distributing pamphlets at local recreational sites and schools in inner-city Oakland. Recruitment targeted African American children with a BMI at or above the 85th percentile. Exclusion criteria were being 8 years of age or younger, being 14 years of age or older, having fasting blood glucose ≥120 mg/dL, having any known metabolic disease, and taking medications known to affect the study outcomes. Parental informed consent was obtained from all subjects, and all protocols were approved by the institutional review boards at the University of California at Berkeley and the University of California at San Francisco. All participants were asked to report to the Children's Hospital and Research Center in Oakland, California, after an overnight fast of at least 12 hours for blood sample collection.

### Anthropometric measurements

Body weight and height were measured to the nearest 0.1 kg and 0.1 cm by using a digital electronic scale (BWB 800, Tanita, Japan) and a portable stadiometer, respectively. BMI, BMI percentiles, and BMI *z *scores were generated by using an age- and sex-specific calculator program (www.cdc.gov/nccdphp/dnpa/growthcharts/resources/sas.htm). Researchers used a plastic, nonelastic measuring tape to measure waist circumference just above the iliac crest with the child in the standing position. Measurements were taken twice and, if a difference of more than 0.4 cm was found between measurements, a third measurement was taken and the mean calculated by using the closest 2 values.

### Biochemical measurements

Fasting blood samples were processed and analyzed by a commercial laboratory (LabCorp, Burlington, North Carolina) for concentrations of HDL-C and triglycerides by using the vertical auto profile cholesterol method (14). Pubertal development on a 5-point scale was assessed by using previously determined serum concentration cutoffs for luteinizing hormone and estradiol (15). Blood glucose was determined by using the hexokinase-peroxidase method (Glucose HK-60 radioimmunoassay, Diagnostic Chemicals, Oxford, Connecticut). Fasting insulin concentrations were determined by using enzyme immunoassay (Linco Research, Inc, St. Charles, Missouri). Fasting blood glucose and insulin values were used to calculate HOMA-IR, defined as fasting blood glucose (mmol/L) × insulin (μIU/mL)/22.5, and used as an index of insulin resistance (13).

### Blood pressure measurements

Blood pressure was measured between 9 am and noon. Measurements were repeated until 2 consecutive systolic and diastolic measurements agreed within 4 and 2 mm Hg, respectively. Measurements were conducted twice at least 3 hours apart, and the second series of measurements was used for analyses. Values were converted to *z *scores (matched for age, height, and sex) by using regression equations developed and reported elsewhere (16).

### MetS incidence

Participants were defined as having MetS if they met the 3 or more of following criteria (4): triglycerides of at least 100 mg/dL, HDL-C less than or equal to 50 mg/dL, fasting blood glucose of at least 110 mg/dL (6.1 mmol/L), waist circumference above the 75th percentile, and systolic or diastolic blood pressure or both above the 90th percentile for age, sex, and height (10). Waist circumference values for the 75th-percentile cutoff, when matched for age and sex, were calculated by using regression equations developed specifically for African American children (17). In some analyses, the blood glucose component of MetS was replaced with values for HOMA-IR, by using a cutoff of 2.5 as suggested previously for assessments of children (18). Throughout this article, the term MetS_glucose_ is used to indicate cases using fasting blood glucose of at least 110 mg/dL as 1 of the 5 components, MetS_HOMA-IR_ is used to indicate cases using HOMA-IR above 2.5 as 1 of the 5 components, and MetS_glucose57_ is used to indicate cases by using fasting blood glucose above the 57th percentile (87.7 mg/dL) as 1 of the 5 components.

### Statistical analyses

A complete set of data was available for 108 of the 125 participants. These data were evaluated for skewedness and, if significant, Dixon's test for outliers was used to identify unusual values. If unusual values were identified, all data for that participant were excluded from further analyses. Using Dixon's test, we excluded data for 3 children, providing a final sample of 105 (45 boys and 60 girls). We analyzed differences in the characteristics of boys and girls, of overweight and obese groups, and of cases compared with noncases by using independent 2-tailed *t *tests following Levene's test for equality of variances for continuous variables and the χ^2^ test for dichotomized variables. Because the term *MetS* is used to describe a single concept and has been defined as a condition comprising at least 3 of 5 interrelated components, correlations among these components, including tests for internal consistency (Cronbach α) were used to compare reliability of fasting blood glucose with HOMA-IR as 1 of the 5 MetS components.

Statistical procedures were performed using SPSS version 16.0 (SPSS, Inc, Chicago, Illinois). Statistical significance was set at *P* < .05.

## Results

Overall, 17% of this sample (9% of boys and 23% of girls) was classified as having MetS_glucose_ because they had values that met the cutoff criteria defined previously by others for 3 or more components (Table 1). In comparison with overweight children (7% of boys and 14% of girls), obese children (10% of boys and 25% of girls) had less favorable values for key health indicators. A total of 9.5% of overweight children (7% of the boys and 14% of the girls) and 19% of obese children (10% of the boys and 24% of the girls) were classified as having MetS_glucose_.

Children who were classified as having MetS_glucose_ had a significantly higher BMI percentile, waist circumference, triglycerides, insulin, systolic blood pressure, and HOMA-IR, and lower HDL-C than those who were negative for MetS_glucose_ (Table 2). Fasting blood glucose concentrations were not significantly different, however, for children with MetS_glucose_. Of the 105 children, only 1 had a fasting blood glucose value that exceeded the cut point of 110 mg/dL. Because this participant had values for 4 components that met the MetS_glucose_ criteria, this blood glucose cutoff, when applied to this population of children, resulted in 100% true positives, 0 false positives, and 100% true negatives (Table 3). The corresponding HOMA-IR value was >11. Although specificity was 100%, sensitivity was 6%, indicating that this component contributed little value for the purpose of diagnosing MetS_glucose_ in this population.

Using HOMA-IR as 1 of the 5 components, we found a 38% prevalence of MetS in this sample of African American children. Replacing the fasting blood glucose component of MetS with HOMA-IR at the cutoff of 2.5 suggested previously for overweight and obese children (18) increased the number of cases from 18 for MetS_glucose_ to 40 for MetS_HOMA-IR_ (Table 2). This HOMA-IR cutoff, when used to assess MetS_HOMA-IR_, resulted in more than 80% true positives and true negatives and less than 20% false positives and false negatives (Table 3). Specificity and sensitivity of HOMA-IR as a MetS component were 83% and 88%, respectively. By using the MetS_HOMA-IR_ cutoffs, we found that 14% of the overweight children (7% of boys and 29% of girls) and 44% of obese children (29% of boys and 53% of girls) were classified as having MetS_HOMA-IR_.

The fasting blood glucose concentration cutoff of 110 mg/dL was at the 99th percentile for this sample, whereas the HOMA-IR cutoff of 2.5 was at the 57th percentile. To more fairly compare the use of fasting blood glucose with HOMA-IR as components of MetS, MetS_glucose57_ was determined by using as the fifth component the 57th percentile for fasting blood glucose concentration in this sample, which was 87.7 mg/dL glucose. This fasting blood glucose concentration, when used to assess MetS_glucose57_ in this population of children, resulted in more than 70% true positives and true negatives, and 28% false positives and 18% false negatives (Table 3). Specificity of the 87.7 mg/dL glucose cutoff as a MetS component was calculated to be 72% and sensitivity was 82%.

Fasting blood glucose concentration was not significantly related to any of the variables included in MetS except for diastolic blood pressure, whereas values for HOMA-IR were significantly related to all MetS variables except for diastolic blood pressure (Table 4a). Glucose concentration, when treated as a dichotomous variable and with cutoffs of either 110 mg/dL or 87.7 mg/dL, was not significantly related to any other dichotomized MetS components with the exception of triglycerides (Table 4b). By contrast, HOMA-IR treated as a dichotomous variable was significantly related to dichotomized waist circumference, HDL-C, and triglycerides.

The intercorrelations among the components were notably lower for the 5 MetS_glucose_ (Cronbach α = 0.424) and MetS_glucose57_ components (0.425) than for the 5 MetS_HOMA-IR_ components (0.548). When other cutoff points for both glucose and HOMA-IR were evaluated, the highest α value observed was for a glucose concentration of 100 mg/dL (0.428) and for HOMA-IR of 2.4 (0.553). Regardless of the glucose concentration cutoffs selected, α values were always lower with the glucose variable (≤0.428) than without it (0.429), indicating that including glucose did not contribute to the reliability of assessing MetS. By contrast, α values were higher with HOMA-IR cutoffs in the 2 to 3 range (0.516-0.553) than without (0.429), indicating that HOMA-IR did contribute to the reliability of MetS assessment.

## Discussion

The prevalence of MetS among children of different ethnicities and backgrounds has been reported in few studies, and the multiple definitions of MetS make it difficult to directly compare population prevalence. Researchers using data from a nationally representative sample of approximately 1,700 adolescents found MetS prevalence to be 13% among 12- to 19-year-old adolescent Mexican Americans, 11% among non-Hispanic whites, and 2.5% among non-Hispanic blacks (4). In our study, using the same MetS criteria, overall prevalence of MetS_glucose_ was 17% (9% of boys, 23% of girls) among a sample of 9- to 13-year-old African American children recruited from inner-city Oakland, California. This finding was lower than the 31% prevalence reported for 12- to 19-year-old adolescents with a BMI in the 85th percentile or higher (4), a difference that may be attributable to the lower age of children in our sample. Our prevalence of 22% for girls was somewhat higher than the 18% prevalence for a sample of predominantly African American, mostly obese, adolescent girls aged 13 to 15 reported by others who used the same MetS criteria (11). The prevalence among girls in our sample was double the prevalence among boys, a finding that is consistent with the sex differences we observed in body fatness (13). Using National Health and Nutrition Examination Survey (NHANES) III data for adolescents aged 12 to 19 — a sample that is more representative of the American civilian population — others have reported a higher overall prevalence among boys than girls (3,4). A follow-up study with a larger sample size will be needed to confirm the sex differences we observed for younger African American overweight and obese children.

The prevalence of MetS_glucose_ was twice as high among obese as among overweight children in our sample (19% and 10%, respectively). In the obese group, 10% of boys and 25% of girls met the criteria for MetS_glucose_ whereas in the overweight group, 7% of boys and 14% of girls met the MetS_glucose_ criteria. Our findings are consistent with analyses of the NHANES III data set for young people aged 12 to 19 years, in which the prevalence of MetS was reported to increase with BMI category (3,4). Thus, our results are similar to previous data yet provide additional information that describes the prevalence of MetS among overweight and obese African American children and suggest the need for additional assessments to further compare boys and girls.

Although fasting glucose concentration has been included by others as a MetS component, our results suggest that insulin resistance may be more reliably used to assess MetS in African American children. In our study, only 1 participant had a fasting blood glucose concentration that exceeded the cut point of 110 mg/dL for MetS. Thus, although highly specific (100%), its use alone would have resulted in a large number (94%) of false negatives and very low sensitivity (6%). Other studies have suggested that, for African American children, insulin resistance is a strong predictor of type 2 diabetes (19), and insulin resistance has always been included previously as a MetS component (20). In our sample, fasting blood glucose and insulin concentrations were not significantly correlated. This is not surprising because hyperinsulinemia is known to developmentally precede the hyperglycemic phase. Both fasting insulin concentrations and HOMA-IR have been shown to be highly correlated with more invasive, exacting, and labor-intensive measures of insulin sensitivity in obese children and adolescents (21). Also, in our sample, fasting glucose concentrations, dichotomized for MetS assessment, were poorly correlated with the other 4 dichotomized components, whereas dichotomized HOMA-IR was significantly correlated. Finally, internal consistency among the MetS components was lower when MetS_glucose_ was included than when MetS_HOMA-IR_ was included. The high levels of specificity (83%) and sensitivity (88%) observed when using the HOMA-IR cutoff of 2.5 as a MetS component suggests that, for African American children, insulin sensitivity should be used instead of glucose concentration to assess children for MetS. This conclusion is further supported by our comparison of HOMA-IR versus glucose when assessed at the same percentile for our sample (ie, the 57th percentile); efforts to identify a glucose concentration that outperformed HOMA-IR as a component were not successful.

Others have attempted to establish the best cutoff value for the HOMA-IR index as a predictor of MetS in children and adolescents. One group concluded that HOMA-IR values "close to 3" seem to be adequate (22), whereas a second group recommended that a cutoff for HOMA-IR of 2.5 be used for obese prepubertal children (18). We chose to use a cutoff of 2.5 for our African American participants for comparison purposes, although Cronbach α was somewhat higher using HOMA-IR 2.4 than 2.5. Our results suggest the necessity of replacing the glucose component with HOMA-IR for MetS diagnosis in this population; the MetS prevalence of 38% in the current sample, determined using HOMA-IR in place of glucose as a component, suggests that this population of children is seriously in need of intervention. A follow-up study is warranted to evaluate MetS prevalence in a larger and more diverse sample of African American children. The optimal HOMA-IR cutoff could also be confirmed in this larger sample.

Limitations of this study include restriction to low-income, inner-city African American children and exclusion of children with a BMI less than the 85th percentile when matched for age and sex. These limitations preclude comparisons among children of different races, ages, and socioeconomic backgrounds, and comparisons with lower BMI children. This is a cross-sectional analysis of data, precluding a cause-and-effect relationship.

In conclusion, among African American boys and girls living in inner-city Oakland, we found that MetS prevalence was 2 to 3 times higher for girls than for boys, even when separated according to the CDC-defined BMI categories, and was twice as high using HOMA-IR (38%) in place of glucose (17%) as a MetS component. Our data suggest that insulin resistance should be used as a MetS component in place of fasting blood glucose, because insulin resistance was more highly correlated with other MetS components, provided fewer false negatives and false positives, and was more sensitive for identifying MetS in this high-risk pediatric population.

## Figures and Tables

**Table 1:** Anthropometric and Hematologic Characteristics of Participants and Differences by Sex and Body Weight, Taking Action Together Study, Oakland, California, 2007

Demographic Characteristics	Sex			Body Weight		

	Boys, Mean (SE) n = 45	Girls, Mean (SE) n = 60	*P* Value^a^	Overweight^b^, Mean (SE) n = 21	Obese^b^, Mean (SE) n = 84	*P* Value^a^
Age, y (SD)	10.6 (1.03)	10.6 (1.18)	.81	10.3 (1.01)	10.7 (1.13)	.16
Pubertal stage (5-point scale)	2.11 (1.48)	3.45 (1.21)	<.001	2.57 (1.66)	2.95 (1.44)	.30
Height, cm	148 (8.93)	151 (9.27)	.06	146 (8.00)	150 (9.36)	.05
Weight, kg	59.3 (18.5)	69.0 (18.8)	.01	45.7 (7.32)	69.6 (18.2)	<.001
BMI percentile	96.0 (4.34)	97.8 (2.70)	.02	91.0 (3.56)	98.5 (1.24)	<.001
BMI, *z *score	1.96 (0.50)	2.21 (0.43)	.007	1.37 (0.21)	2.29 (0.32)	<.001
WC, cm	84.5 (14.8)	93.0 (14.9)	.005	71.4 (7.11)	93.9 (13.5)	<.001
WC >75th percentile^c^ % of sample	96	95	.90	76	100	<.001^d^
HDL-C, mg/dL	57.4 (13.2)	52.5 (11.4)	.04	63.0 (14.5)	52.5 (10.9)	<.001
Triglycerides, mg/dL	63.4 (27.1)	76.3 (25.2)	.01	60.0 (23.7)	73.5 (26.8)	.04
Fasting glucose, mg/dL	87.6 (5.94)	88.3 (15.3)	.76	87.4 (6.71)	88.1 (13.2)	.82
Insulin, μIU/mL	8.49 (5.08)	16.3 (12.3)	<.001	6.84 (4.02)	14.5 (11.2)	<.001
HOMA-IR^e^	1.86 (1.17)	3.57 (2.68)	<.001	1.49 (0.93)	3.71 (2.44)	<.001
sBP, mm/Hg	106 (8.79)	105 (7.38)	.77	102 (6.26)	106 (8.22)	.06
dBP, mm/Hg	62.5 (8.40)	62.5 (7.83)	.99	59.6 (5.90)	63.2 (8.37)	.07
sBP, *z *score	0.02 (0.74)	−0.06 (0.72)	.58	−0.22 (0.56)	0.02 (0.75)	.17
dBP, *z *score	0.01 (0.69)	−0.03 (0.72)	.81	−0.22 (0.50)	0.04 (0.74)	.14
Metabolic syndrome,^f^ n (% of sample) with glucose ≥110 mg/dL	4 (8.9)	14 (23.3)	.05^d^	2 (9.5)	16 (19.0)^f^	.30^d^

Abbreviations: SE, standard error; BMI, body mass index; WC, waist circumference; HDL-C, high-density lipoprotein cholesterol; HOMA-IR, homeostasis model assessment of insulin resistance; sBP, systolic blood pressure; dBP, diastolic blood pressure.

a Differences determined using 2-tailed *t* test following Levene's test for equality of variances (with exception noted in footnote c).

b Overweight is defined as BMI >85 to <95th percentile; obese is defined as BMI ≥95th percentile matched for age and sex.

c Waist circumference percentiles calculated using regression equations developed by Fernandez et al (17) for African American children with adjustments for age and sex.

d Difference determined using χ^2^ test.

e HOMA-IR, defined as fasting blood glucose (mmol/L) × insulin (μIU/mL)/22.5, and used as an index of insulin resistance. Cutoffs for defining metabolic syndrome in children taken from de Ferranti et al (4).

f Of those children with BMIs ≥95th percentile, the proportion of girls (29%) compared with boys (18%) that met the criteria for having metabolic syndrome was not significantly different (χ^2^ test).

**Table 2:** Anthropometric and Hematologic Characteristics of Participants With and Without Metabolic Syndrome Using 2 Sets of Criteria,^a^ Taking Action Together Study, Oakland, California, 2007

Participant Characteristics	MetS_glucose_ Status			MetS_HOMA-IR_ Status		

	Negative, Mean (SEM) n = 87	Positive, Mean (SEM) n = 18	*P* Value^b^	Negative, Mean (SEM) n = 65	Positive, Mean (SEM) n = 40	*P* Value^b^
Sex (0 = girls; 1 = boys)	0.47 (0.50)	0.22 (0.43)	.05	0.54 (0.50)	0.25 (0.44)	.004
Pubertal stage (5-point scale)	2.80 (1.44)	3.22 (1.44)	.28	2.66 (1.45)	3.22 (1.49)	.06
BMI, *z *score	2.07 (0.49)	2.29 (0.37)	.04	1.97 (0.49)	2.31 (0.37)	<.001
Waist circumference, cm	87.9 (15.5)	96.3 (13.1)	.03	85.2 (14.3)	96.1 (14.8)	<.001
HDL-C, mg/dL	56.7 (12.5)	44.5 (4.18)	<.001	60.5 (11.6)	44.9 (5.65)	<.001
Triglycerides, mg/dL	62.8 (20.5)	109.0 (19.1)	<.001	60.1 (19.6)	88.1 (27.8)	<.001
Fasting glucose, mg/dL	86.6 (5.78)	94.9 (26.0)	.12	86.2 (5.78)	90.9 (18.1)	.06
Fasting insulin, μIU/mL	12.0 (10.5)	17.5 (10.4)	.04	9.83 (10.3)	18.1 (9.04)	<.001
HOMA-IR	2.61 (2.21)	4.08 (2.51)	.01	2.09 (2.13)	4.04 (2.12)	<.001
sBP, z score	−0.10 (0.69)	0.31 (0.80)	.03	−0.18 (0.65)	0.22 (0.77)	.004
dBP, z score	−0.05 (0.67)	0.18 (0.86)	.20	−0.08 (0.65)	0.09 (0.78)	.25

Abbreviations: BMI, body mass index; HDL-C, high-density lipoprotein cholesterol; HOMA-IR, homeostasis model assessment of insulin resistance; sBP*z*, systolic blood pressure *z *score; dBP*z*, diastolic blood pressure *z *score.

a Defined as a glucose concentration cutoff of ≥110mg/dL (MetS_glucose_) or a HOMA-IR cutoff of ≥2.5 (MetS_HOMA-IR_).

b Differences determined using 2-tailed *t* tests following Levene's test for equality of variances with the exception that χ^2^ test was used for the dichotomous variable "sex."

**Table 3:** Reliability of Glucose Compared with HOMA-IR as 1 of the 5 Components of Metabolic Syndrome, Taking Action Together Study, Oakland, California, 2007

Cutoffs	Positives			Negatives			Specificity,^f^%	Sensitivity,^g^%

	No. of Cases^a^	True,^b^n (%)	False,^c^n (%)	No. of Cases,^a^n	True,^d^n (%)	False,^e^n (%)		
Glucose ≥110 mg/dL^h^	18	1 (100)	0	87	87 (100)	17 (94)	100	6
HOMA-IR ≥2.5^i^	40	35 (88)	11 (17)	65	54 (83)	5 (13)	83	88
Glucose ≥87.7 mg/dL^i^	33	27 (82)	20 (28)	72	52 (72)	6 (18)	72	82

Abbreviations: HOMA-IR, homeostasis model assessment of insulin resistance; HDL-C, high-density lipoprotein cholesterol; MetS, metabolic syndrome.

a For all conditions, 4 components were evaluated: waist circumference, HDL-C, triglycerides, and blood pressure. A fifth component (glucose concentration or HOMA-IR value) was evaluated at the values indicated in the cutoffs column.

b True positives, % = [(number of positive cases for which glucose or HOMA-IR values exceeded the indicated cutoff) / (total number of positive cases)] X 100.

c False positives, % = [(number of negative cases for which glucose or HOMA-IR values exceeded the indicated cutoff) / (total number of negative cases)] X 100.

d False positives, % = [(number of negative cases for which glucose or HOMA-IR values exceeded the indicated cutoff) / (total number of negative cases)] X 100.

e False negatives, % = [(number of positive cases for which glucose or HOMA-IR values did not exceed the indicated cutoff) / (total number of positive cases)] X 100.

f Specificity = [(number of true negatives) / (number of true negatives + number of false positives)] X 100.

g Sensitivity = [(number of true positives) / (number of true positives + number of false negatives)] X 100.

h Glucose concentration cutoff for MetS as recommended by de Ferranti et al (4).

i HOMA-IR, defined as fasting blood glucose (mmol/L) × insulin (μIU/mL)/22.5. HOMA-IR cutoff for MetS as recommended by Madeira et al (18). In this sample, 57% of participants had HOMA-IR <2.5 and 57% had fasting blood glucose concentrations <87.7 mg/dL.

**Table 4:** Pearson's Correlations and Significance Between Values for Blood Glucose or Insulin Resistance and Other Metabolic Risk Factors (n = 105), Taking Action Together Study, Oakland, California, 2007


Table 4a. Correlations, treating each component as a continuous variable.				

MetS Component	Glucose, mg/dL		HOMA-IR	

	*r*	*P* Value	*r*	*P* Value
Waist circumference, cm	0.13	.19	0.51	<.001
HDL-C, mg/dL	−0.10	.33	−0.27	.006
Triglycerides, mg/dL	0.19	.06	0.26	.007
sBP, z score	−0.40	.69	0.21	.03
dBP, z score	−0.20	.05	0.14	.15
Insulin, μIU/mL	0.06	.57	0.98	<.001


Table 4b. Correlations, treating each component as a dichotomous variable.						

MetS Component	Glucose ≥110 mg/dL^a^		Glucose, 57th Percentile or≥87.7 mg/dL^b^		HOMA-IR,57th Percentile or ≥2.5^b^	

	*r*	***P* Value**	*r*	*P* Value	*r*	*P* Value
Waist circumference, cm^c^	0.02	.82	0.02	.83	0.20	.04
HDL-C^d^	0.10	.29	0.08	.42	0.37	<.001
Triglycerides^e^	0.21	.03	0.17	.08	0.28	.003
BP^f^	−0.03	.76	0.13	.17	0.07	.46

Abbreviations: HOMA-IR, homeostasis model assessment of insulin resistance; HDL-C, high-density lipoprotein cholesterol;  sBP; systolic blood pressure; dBP, diastolic blood pressure; BP, blood pressure.

a Glucose concentration cutoff at ≥110 mg/dL: 0 = below cutoff; 1 = above cutoff.

b HOMA-IR cutoff of ≥2.5 was at the 57th percentile for this population (0 = below cutoff, 1 = above cutoff). The corresponding 57th percentile glucose concentration in this population was 88 mg/dL.

c Waist circumference cutoff for MetS was >75th percentile when matched for age, sex and race: 0 = below cutoff; 1 = above cutoff.

d HDL-C cutoff for MetS was <50 mg/dL: 0 = above cutoff; 1 = below cutoff.

e Triglycerides cutoff for MetS was ≥100 mg/dL: 0 = below cutoff; 1 = above cutoff.

f BP cutoff for MetS: sBP and/or dBP >90th percentile when matched for age, sex, and height: 0 = below cutoff; 1 = above cutoff.
